# New approach methods to improve human health risk assessment of thyroid hormone system disruption–a PARC project

**DOI:** 10.3389/ftox.2023.1189303

**Published:** 2023-05-17

**Authors:** Louise Ramhøj, Marta Axelstad, Yoni Baert, Ana I. Cañas-Portilla, Frédéric Chalmel, Lars Dahmen, Antonio De La Vieja, Bertrand Evrard, Ann-Cathrin Haigis, Timo Hamers, Kim Heikamp, Henrik Holbech, Patricia Iglesias-Hernandez, Dries Knapen, Lorna Marchandise, Jane E. Morthorst, Nikolai Georgiev Nikolov, Ana C. V. E. Nissen, Michael Oelgeschlaeger, Kostja Renko, Vera Rogiers, Gerrit Schüürmann, Evelyn Stinckens, Mette H. Stub, Monica Torres-Ruiz, Majorie Van Duursen, Tamara Vanhaecke, Lucia Vergauwen, Eva Bay Wedebye, Terje Svingen

**Affiliations:** ^1^ Research Group for Molecular and Reproductive Toxicology, National Food Institute, Technical University of Denmark, Kgs. Lyngby, Denmark; ^2^ Department In Vitro Toxicology and Dermato-cosmetology (IVTD), Vrije Universiteit Brussel, Jette, Belgium; ^3^ Environmental Toxicology Unit from National Center for Environmental Health (CNSA), Endocrine Tumor Unit from UFIEC, Instituto de Salud Carlos III (ISCIII), Madrid, Spain; ^4^ Univ Rennes, Inserm, EHESP, Irset (Institut de Recherche en Santé, Environnement et Travail), Rennes, France; ^5^ Department Experimental Toxicology and ZEBET, German Centre for the Protection of Laboratory Animals (Bf3R), German Federal Institute for Risk Assessment (BfR), Berlin, Germany; ^6^ Zebrafishlab, Veterinary Physiology and Biochemistry, Department of Veterinary Sciences, University of Antwerp, Wilrijk, Belgium; ^7^ Amsterdam Institute for Life and Environment (A-LIFE), Vrije Universiteit Amsterdam, Amsterdam, Netherlands; ^8^ Centre for Health Protection (GZB), National Institute for Public Health and the Environment (RIVM), Bilthoven, Netherlands; ^9^ Department of Biology, University of Southern Denmark, Odense, Denmark; ^10^ Group for Chemical Risk Assessment and GMO, National Food Institute, Technical University of Denmark, Kgs. Lyngby, Denmark; ^11^ UFZ Department of Ecological Chemistry, Helmholtz Centre for Environmental Research, Leipzig, Germany

**Keywords:** PARC, endocrine disruption, thyroid disruption, non-animal test methods, regulatory toxicology, adverse outcome pathways, chemicals

## Abstract

Current test strategies to identify thyroid hormone (TH) system disruptors are inadequate for conducting robust chemical risk assessment required for regulation. The tests rely heavily on histopathological changes in rodent thyroid glands or measuring changes in systemic TH levels, but they lack specific new approach methodologies (NAMs) that can adequately detect TH-mediated effects. Such alternative test methods are needed to infer a causal relationship between molecular initiating events and adverse outcomes such as perturbed brain development. Although some NAMs that are relevant for TH system disruption are available–and are currently in the process of regulatory validation–there is still a need to develop more extensive alternative test batteries to cover the range of potential key events along the causal pathway between initial chemical disruption and adverse outcomes in humans. This project, funded under the Partnership for the Assessment of Risk from Chemicals (PARC) initiative, aims to facilitate the development of NAMs that are specific for TH system disruption by characterizing *in vivo* mechanisms of action that can be targeted by *in embryo/in vitro/in silico/in chemico* testing strategies. We will develop and improve human-relevant *in vitro* test systems to capture effects on important areas of the TH system. Furthermore, we will elaborate on important species differences in TH system disruption by incorporating non-mammalian vertebrate test species alongside classical laboratory rat species and human-derived *in vitro* assays.

## 1 Introduction

Within current European regulatory testing frameworks, chemical compounds that can cause adverse effects *in vivo* through thyroid hormone (TH) system disruption are primarily assessed in laboratory rodents. Assessments typically focus on gross effects on the thyroid gland itself, including changes in weight or histopathology, or by measuring levels of circulating THs (ECHA/EFSA, 2018). Although these measurements are informative, they are inadequate when it comes to identifying the broad suite of potential TH system disruptors (Noyes et al., 2019; Couderq et al., 2020; Kortenkamp et al., 2020). For instance, it is recognized that many potential TH system disruptors can act via a number of different mechanisms that are not detected by analyzing the thyroid gland itself, nor by measuring systemic levels of the THs triiodothyronine (T3) and thyroxine (T4) (Noyes et al., 2019; Gilbert et al., 2020). This is because changes in, for instance, deiodinase activity or membrane transport of THs, will not necessarily affect circulating levels of TH, but could still have a large impact on TH availability and action in target tissues (Landers and Richard, 2017; Gilbert et al., 2020; Köhrle and Frädrich, 2022). Lastly, TH-mediated adverse effects in target tissues peripheral to the thyroid gland are not addressed by current test methods, including TH-mediated developmental neurotoxicity.

By early 2023, more than 22,000 unique industrial chemicals were registered under the European REACH regulation (Registration, Evaluation, Authorisation and restriction of Chemicals) (Directorate-General for Environment, 2023). The REACH standard information requirements are based on the tonnage level of combined import and production per registrant, but the same chemical can be imported or produced by several different registrants. Based on today’s information requirements, this means that *in vivo* tests that aim to determine whether chemicals possess TH system disrupting properties in mammals are not performed for chemicals at the 1–10 tonnes/year level (per registrant). From 10 tonnes and above, endpoints and biomarkers such as TH concentrations and thyroid histopathology are included in some rodent tests but, as already discussed, these are insufficient in identifying all TH system disruptors. Although pesticides and biocides (under the plant protection products or biocidal products regulations) are subject to much stronger testing requirements despite low tonnage levels, the tests, biomarkers, and endpoints are the same as for REACH registered chemicals. There is thus a pressing need to establish additional measurable endpoints or biomarkers for testing purposes including new approach methods.

**FIGURE 1:**
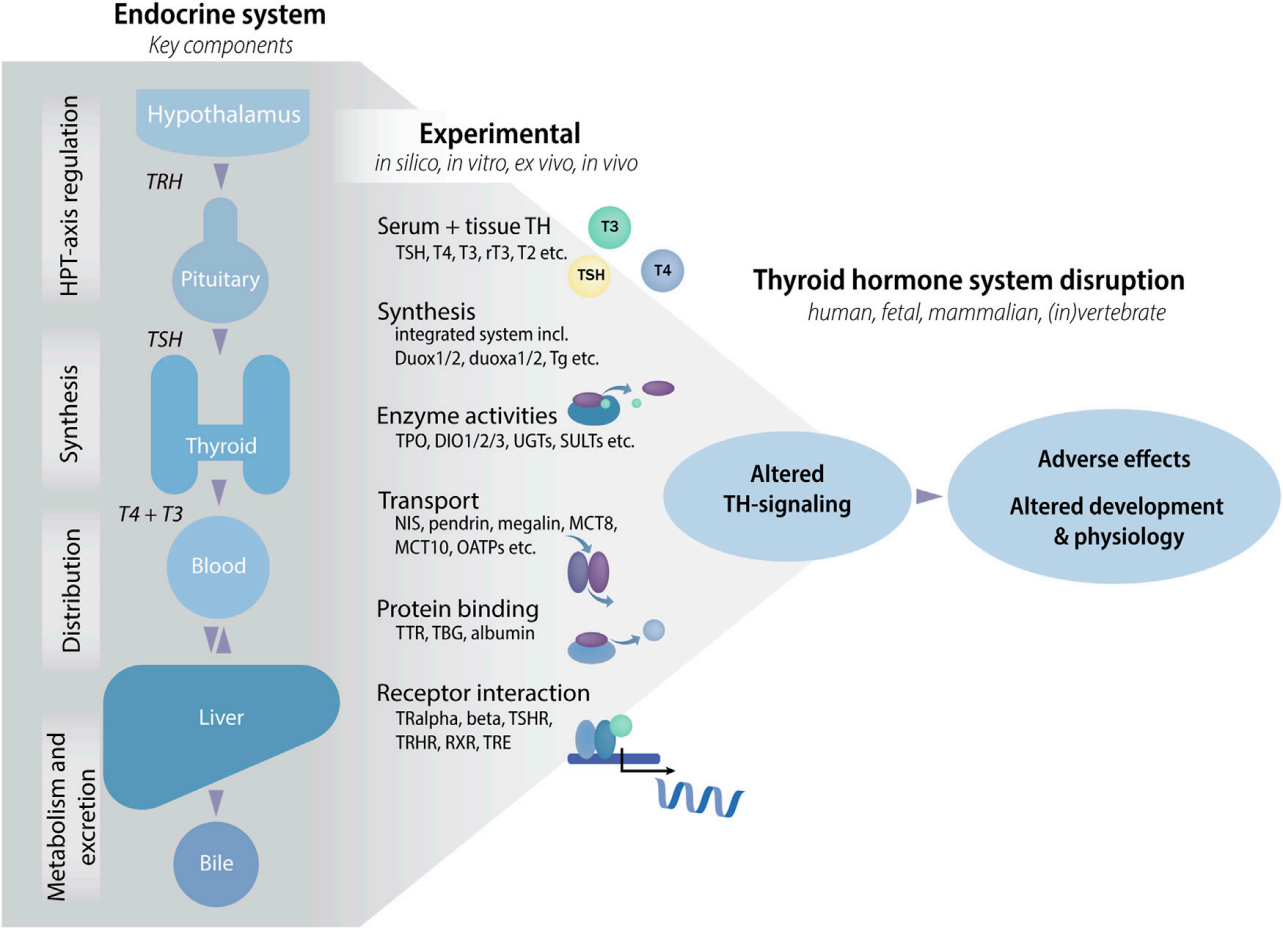
The thyroid hormone (TH) system and tests to assess chemical effects that may lead to altered TH-signaling and adverse effects. The TH system has many components and molecules that can be targeted by environmental chemicals in one and/or all tissues. Targets can be grouped into classes according to the type of assay that can be used to test for potential effects of chemicals, e.g., by developing enzyme activity assays where each assay tests the activity of one enzyme, TPO, DIO1, DIO2, DEHAL1 etc. *In vivo,* each interaction may lead to altered TH-signaling with potential adverse effects on development and physiology. Abbreviations: DEHAL1: iodothyrosine dehalogenase, DIO: deiodinase, HPT: hypothalamic-pituitary-thyroid, MCT: monocarboxylate transporter, NIS: Sodium/Iodide Symporter, OATPs: organic-anion-transporting polypeptides, rT3: reverse T3, RXR: retinoid X receptor, SULTs: sulfotransferases, T2: 3,5-diiodo-L-thyronine, T3: triiodothyronine, T4: thyroxine, TBG: thyroxine-binding globulin, Tg: thyroglobulin, TH: thyroid hormone, TPO: thyroperoxidase, TR: thyroid receptor, TRE: thyroid response element, TRH: thyrotropin releasing hormone, TSH: thyroid stimulating hormone, TSHR: thyroid stimulating hormone receptor, TTR: transthyretin, UGTs: Uridine 5′-diphospho-glucuronosyltransferases.

Decades of research into the hypothalamic-pituitary-thyroid (HPT) axis, and the biological functions of the THs, has identified a complex network of endocrine regulation and highlighted multiple entry points for possible disruption by chemical substances. These include TH synthesis, transport and receptor binding, as well as local tissue uptake and metabolism (Figure 1) (OECD, 2014; Noyes et al., 2019; Gilbert et al., 2020). The potential molecular initiating events (MIEs) for TH system disruptors can be tested for by various new approach methodologies (NAMs), including *in silico* approaches, *in chemico* and *in vitro* assays as well as information from chemical exposure for hazard assessment (European Chemicals Agency, 2016). Indeed, a number of newly developed NAMs have already shown great promise for future implementation in international test guideline programmes and hence are currently in the process of regulatory validation within the framework of the Organization for Economic Co-operation and Development (OECD) (Joint Research Centre, 2023). However, even with these new additions to a broader testing battery of methods relevant for assessing TH system disruption, not all critical steps between a MIE and an adverse outcome (AO) in adverse outcome pathways (AOPs) are covered.

A major challenge in developing new NAMs for TH system disruption is the lack of fundamental biological knowledge of key aspects of TH-mediated developmental processes. Thus, current efforts aimed at improving our capacity to safeguard human and ecological health from TH system disrupting chemicals should include efforts to better understand the complex biology of toxicological effects *in vivo.* And these efforts should leverage the knowledge we already possess to devise a broad NAM-based screening battery capable of identifying key events (KEs) of TH-mediated causal pathways. In this project, that we are performing under the European Union (EU)-funded Partnership for the Assessment of Risk from Chemicals (PARC, 2023) programme (Marx-Stoelting et al., 2023; PARC), we aim to provide both novel biological knowledge of how THs may regulate development and how TH system disruptors can perturb these processes, in order to establish and develop NAMs to be incorporated in regulatory decision making for the identification of TH system disruptors.

## 2 Background

### 2.1 Thyroid hormone system disruption and the developing organism

THs are involved in the differentiation, growth, and function of virtually all vertebrate tissues and organs (Yen, 2001). They play distinct roles throughout life and exert specific actions in a spatiotemporal manner through both systemic and local regulation of TH signaling. This is achieved by a fine-tuned regulation of an intricate network of TH synthesis, feedback mechanisms, serum distribution, membrane transporters, metabolizing enzymes, receptors and more (Zoeller et al., 2007; Ortiga-Carvalho et al., 2016). The different components all work in concert to deliver THs to target cells where they regulate gene expression through interactions with nuclear TH receptors (TRs) or through non-genomic mechanisms (Ortiga-Carvalho et al., 2016; Taylor and Heyland, 2022).

TH action is critical for the development of the central nervous system where THs help regulate crucial events such as neuronal differentiation, migration, synaptogenesis and myelination. Deficiency in THs during these events can thus have profound and irreversible effects on the developing brain. Worryingly, many environmental chemicals have been shown to act as TH system disruptors and can thus potentially affect brain development with significant consequences for life-long cognition (Gilbert et al., 2020; Marty et al., 2022). In this context, it is also important to consider the regulatory networks that finetune TH action in complex organisms. For instance, THs can act directly on the developing liver and control processes such as lipid metabolism, but the liver itself may also modify systemic TH regulation. Deciphering these mechanisms of effects and function will be critical if we are to faithfully capture potential TH system disrupting chemicals by use of alternative test method batteries.

### 2.2 Pregnant women are uniquely sensitive to thyroid hormone system disruption

**FIGURE 2:**
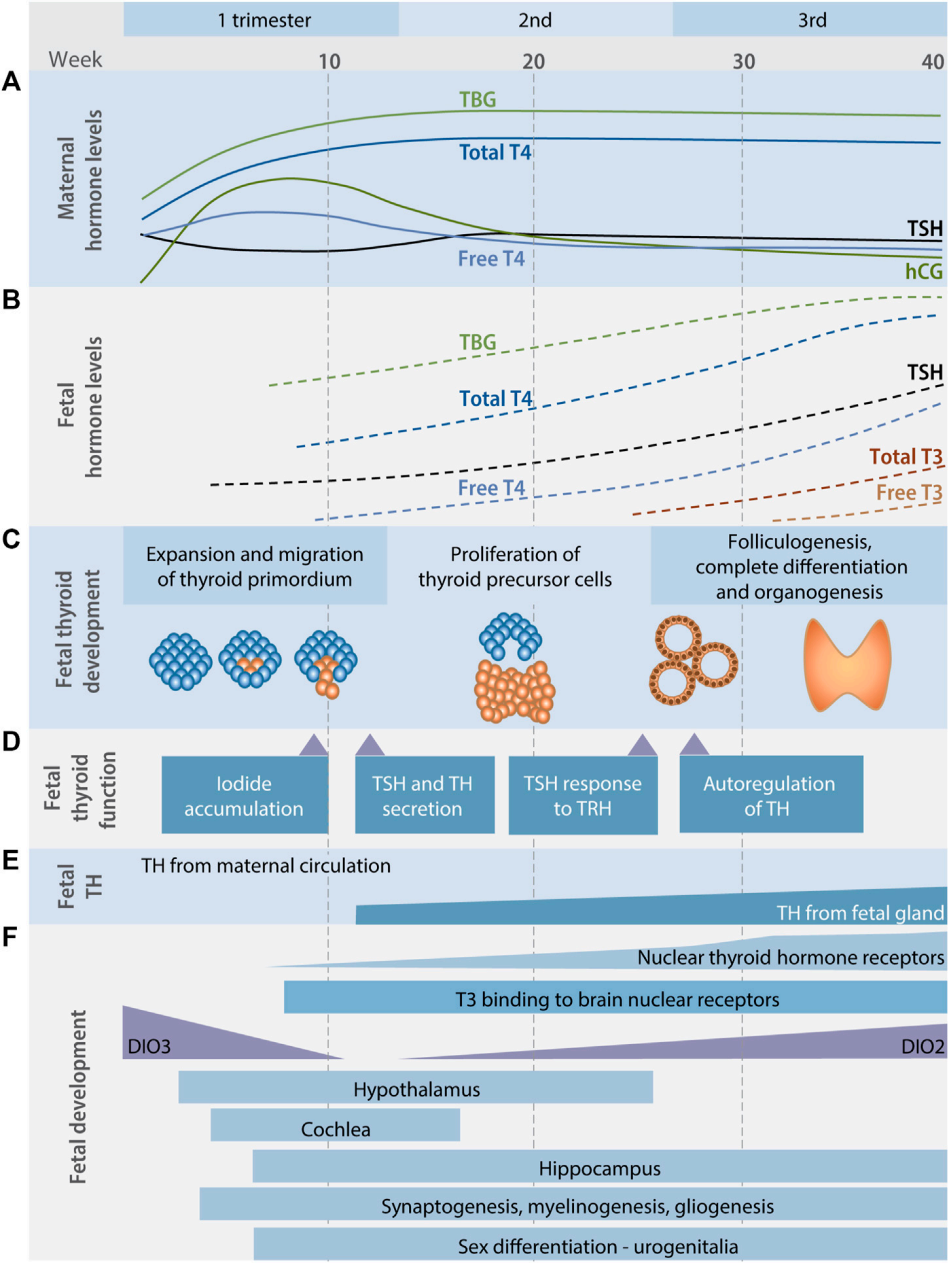
The thyroid hormone system and fetal development. Maternal **(A)** and fetal **(B)** TH levels during human pregnancy. In this period the requirement for iodine in the mother’s diet increases considerably, as does the net demand for TH production by the maternal thyroid gland. Before the onset of fetal thyroid function the fetus is critically dependent on TH transferred from the maternal to the fetal circulation. Timing of ontogeny **(C)**, function and autoregulation **(D)** of the fetal thyroid gland during pregnancy. **(E)** Maternal TH fully supply fetal requirements until fetal TH secretion begin at around 15 weeks of gestation after which fetal TH levels come from both the maternal and the fetal thyroid gland. **(F)** Timing of some TH mediated neurodevelopmental processes in the fetal brain. Early neuronal proliferation and migration is dependent on maternal TH in the first trimester of pregnancy. Also in the first trimester, inactivating type 3 deiodinase (DIO3) enzyme expression drops and development of the thyroid gland begins. In the second and third trimesters, the brain continues to develop, now increasingly relying on T4 produced by both the fetus and the mother. As the fetal hypothalamic-pituitary axis develops, a surge in thyroid-stimulating hormone (TSH) secretion occurs, initiating fetal TH production, expression of the activating enzyme deiodinase type 2 (DIO2), and increasing thyroid hormone nuclear receptors occupancy by T3. DIO: deiodinase, hCG: human chorionic gonadotropin, T3: triiodothyronine, T4: thyroxine, TBG: thyroxine-binding globulin, TH: thyroid hormone, TRH: thyrotropin releasing hormone, TSH: thyroid stimulating hormone, (Burrow et al., 1994; Howdeshell, 2002; López-Márquez et al., 2021).

The developing mammalian fetus does not synthesize THs during early stages of gestation and is therefore dependent on maternal supply at critical stages of neuronal development (Figure 2). In humans, the fetal thyroid gland starts producing THs by the second trimester, but maternal supply continues to be important throughout gestation until birth (Morreale de Escobar et al., 2004). Consequently, fetal brain development is dependent on maternal thyroid function, something that is evident from numerous epidemiological studies linking low maternal T4 levels to child neurodevelopmental effects (Henrichs et al., 2010; Román et al., 2013; Ghassabian et al., 2014; Modesto et al., 2015; Gyllenberg et al., 2016; Korevaar et al., 2016). From the second trimester of pregnancy, a correct iodine supply from the mother to the fetus is also essential, so that the fetus can produce its own THs (De La Vieja and Santisteban, 2018). Therefore, amongst other things, the presence of membrane transporters of both iodide and THs in the placenta throughout pregnancy is essential to maintain the correct balance of mother-fetus TH levels.

During pregnancy, both total and free T4 levels increase during the first trimester to reach a steady state that is maintained for the remainder of the gestational period. Although these absolute changes to TH levels are relatively small, there is a net loss of THs and an increase in hormone production by the thyroid gland. This is because there is a net increase in plasma volume as the fetus grows, so that more TH must be produced to keep steady state conditions, but also due to an increase in renal excretion of iodine that potentially reduces available iodine in the thyroid gland. Furthermore, during the first trimester, human chorionic gonadotropin (hCG) stimulates TH production in the thyroid gland and the presence of estrogen causes an increase in thyroxine-binding globulin (TBG). Later in gestation there are also changes to peripheral TH metabolism (Howdeshell, 2002; Morreale de Escobar et al., 2004). Combined, all these factors increase the net demand for TH synthesis in the thyroid gland; demands that can be met by a healthy and robust TH system, but not necessarily by a compromised TH system.

### 2.3 Identification of thyroid hormone system disrupting compounds

Current chemical legislation in the EU mandate the testing of potential endocrine disrupting properties for pesticides, biocides and REACH-regulated chemicals produced at high tonnage levels. These assessments typically include evaluation of estrogenic, androgenic, thyroid, and steroidogenesis (EATS) modalities using various test assays and animal toxicity studies in a tiered process (ECHA/EFSA, 2018; OECD, 2018). Of the five tiers, the first two can be performed animal-free, whereas the last three tiers require animal testing. In many ways, the EATS modalities have, to a large extent, defined the types of assays that are used to test for endocrine disruption. As already discussed, a challenge with respect to the thyroid (T) modality is that current OECD test methods remain inadequate when it comes to identifying TH system disruption and its adverse effects on the developing organism (Gilbert et al., 2020; Kortenkamp et al., 2020); not only because we do not have an adequate battery of NAMs to capture all potential MIEs and KEs, and lack sensitive *in vivo* tests for TH-mediated adversity, but also because current regulation requires *in vivo* evidence to support indicative NAMs data. Notably, the REACH §44 (1) risk concept mentions explicitly structural similarity and the relevance of transformation products, thus pointing to *in silico* (e.g., read-across, ligand-receptor docking) and *in chemico* (compound transformation to active or inactive metabolites) approaches to support compound evaluation, but the regulatory frameworks still have a way to go in incorporating non-animal test data for chemical regulation.

### 2.4 Current state of AOPs for thyroid hormone system disruption

TH system disruption has been identified as one of the priority areas for AOP and integrated approaches for testing and assessment (IATA) development within PARC. In this context, there is close interaction with the work on AOP development for endocrine and metabolic disruption (PARC Task 5.3.2) and the development of IATAs for endocrine disruption (PARC Task 6.1.1). All experimental data on TH system disruption that are generated in PARC are being mapped to the AOP network, either for improving or expanding existing AOPs, or for the development of new AOPs. The PARC endocrine disruption AOP and IATA development processes are closely aligned to ensure that the methods that are considered for inclusion in the IATAs are supported by KE descriptions of well-developed AOPs. The development of TH system disruption IATAs for both human and environmental health has been initiated, and since these IATAs are based on the conservation of pathways across vertebrates, envisioned to share a number of early KE, the work on assay development and cross-species comparison and extrapolation will be highly relevant to support these activities.

At time of writing this report, there are around 30 AOP pages for TH system disruption already available in the AOP-Wiki, together including 50 linear AOPs. A limited number of AOPs have been developed to the level where they have been endorsed by the OECD Working Party on Hazard Assessment and the Working Group of National Coordinators of the Test Guidelines Programme (WPHA/WNT). This includes the AOP initiated by inhibition of thyroperoxidase (TPO) and leading to adverse neurodevelopmental outcomes in mammals (Crofton et al., 2019) and the AOP leading from inhibition of the Na+/I- symporter (NIS) to learning and memory impairment in mammals (Rolaki et al., 2019). Five AOPs linking TPO and deiodinase inhibition to impaired swim bladder inflation in fish have recently been endorsed (Vergauwen et al., 2022a; Vergauwen et al., 2022b; Vergauwen et al., 2022c; Vergauwen et al., 2022d; Vergauwen et al., 2022e). A multitude of other AOPs describing TH system disruption with applicability to different taxa are in varying stages of development. When combining these AOPs in one large AOP network, a cross-species AOP network emerges (Knapen et al., 2018; Noyes et al., 2019; Knapen et al., 2020).

As a first step in using this AOP network to support cross-species extrapolation and sharing of knowledge between human and environmental health, the taxonomic domain of applicability of the MIEs and the AOs in the network was investigated in the context of altered TH levels. This effort, which was part of the Horizon 2020 ERGO project (Holbech et al., 2020), was based on both an analysis of target conservation (for the MIEs) using the US Environmental Protection Agency’s Sequence Alignment to Predict Across Species Susceptibility (SeqAPASS) tool, and empirical evidence from the literature for the MIEs and AOs. While most AOPs typically are initially developed for one species or taxonomic group, evidence was found that multiple molecular initiating events and adverse outcomes are in fact applicable across different vertebrate taxa. This analysis provides a basis for developing case studies to investigate responses to TH system disrupting chemicals across species, and the development of approaches for AOP network-based cross-species extrapolation.

## 3 The project: to improve and develop NAMs for human health-relevant risk assessment of thyroid hormone system disruptors

**FIGURE 3:**
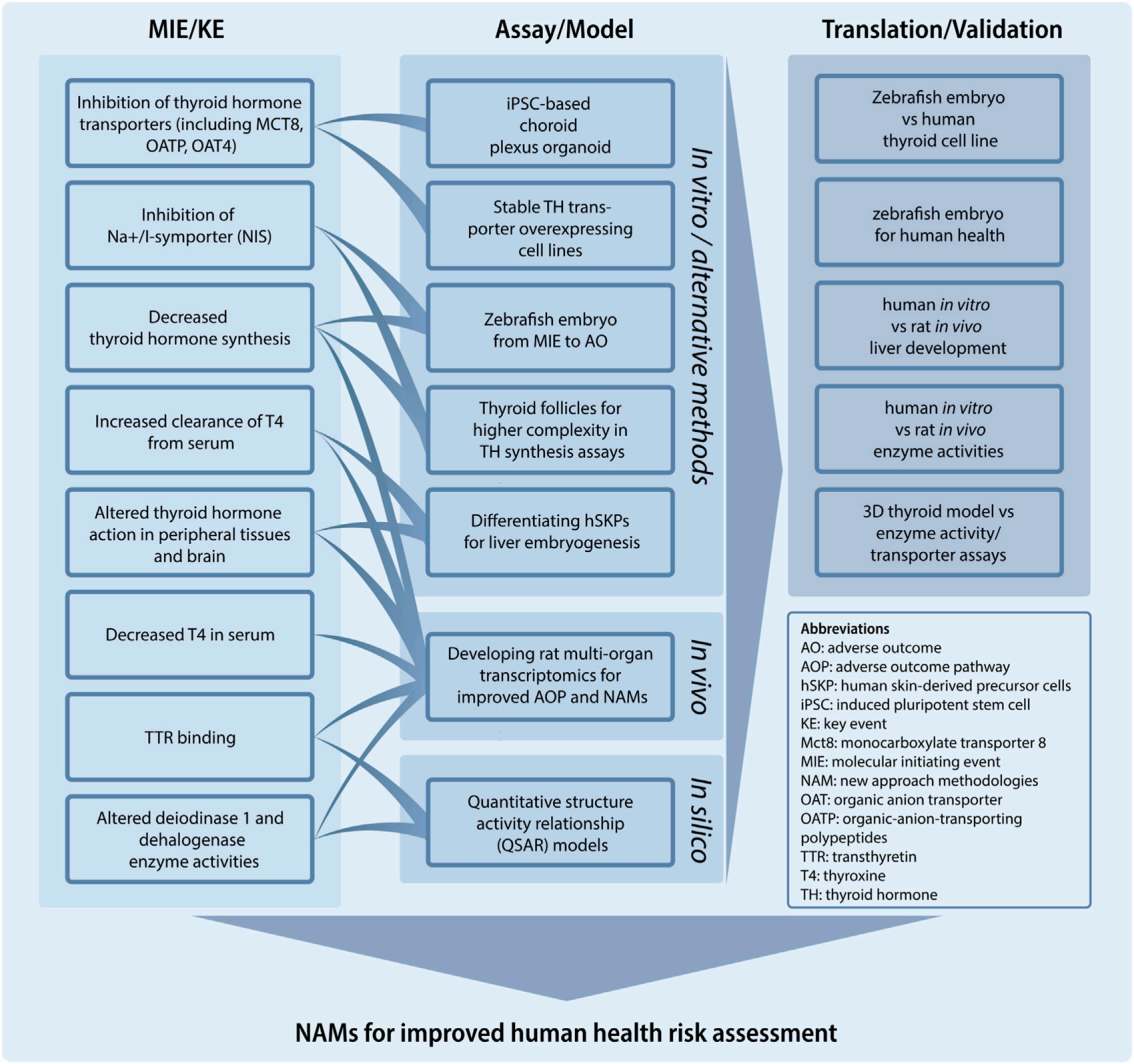
The project: New approach methodologies (NAMs) for improved human health risk assessment. Building upon the adverse outcome pathway (AOP)-framework the project develops NAMs relating to specific molecular initiating events (MIE) and key events (KE) for thyroid hormone system disruption. Some of these relations are shown. The project also includes a number of activities to translate and/or validate assays, including leveraging data from zebrafish embryos to inform on human health and *in vitro* to *in vivo* translation.

Despite previous activities at the OECD level and in various EU initiatives on the identification, development and evaluation of *in vitro* methods addressing the thyroid hormone system (OECD, 2014), we still lack key knowledge about the many MIEs and downstream KEs that can lead to TH-mediated AOs. To address this challenge, this specific project under the PARC programme, aims to provide new mechanistic knowledge that will facilitate NAM development (Figure 3). Specifically, we will address key characteristics of TH system disruption such as TH system regulation and specific points of vulnerability to chemical disruption. This will include direct effects on the developing brain by diminished TH levels, but also effects mediated through other targets such as the liver. Thus, direct versus indirect perturbation of the TH axis by exogenous chemical substances will be scrutinized by use of sophisticated NAMs that build on, for instance, human stem cell-based *in vitro* assays. We will develop and mature *in vitro* and *in silico* tests to capture effects on key components of the TH system, such as TH transport across physiologic barriers. Importantly, we will include the characterization of evolutionary conservation of the TH system axis between rats and humans, but also mammalian and non-mammalian vertebrates (e.g., fish and amphibians) and conserved elements of the thyroid-like system that exist in some invertebrate phyla (e.g., mollusks) to allow for additional NAMs that can inform on human health hazards. The project will focus on the following three areas and align activities with the AOP framework to fill essential data gaps necessary for improved risk assessment regimens for TH system disruptors.

### 3.1 From *in vivo* rodent toxicogenomics to human-relevant NAMs

We will address considerable knowledge gaps about how TH system disruptors act in the developing organism. It still remains poorly understood how different MIEs cause effects throughout the organism. This is partly because THs exert specific spatiotemporal functions during development; functions that are not well characterized. Another uncertainty relates to how chemical exposure, TH signaling, and developmental processes interact both systemically and locally. It is recognized that disrupted hormone concentrations at the systemic level can cause varying effects locally, also for TH system disruption, as recently exemplified in rats exposed to the perfluorinated compound PFOS (Davidsen et al., 2022). This means that we need to carefully consider the mechanisms or effects that we are targeting with NAMs when designing non-animal test methods for predictive toxicology purposes. We will leverage *in vivo* rat toxicity studies with multi-organ RNA-seq approaches (e.g., brain, liver, thyroid gland, heart) to decipher modes of action and characterize relationships between different MIEs, their downstream KEs and ultimately AOs. This will provide knowledge needed to pinpoint future NAMs that are necessary to cover more completely the various MIEs, pathways and effects that TH system disruption may have on the intact organism.

### 3.2 In vitro assays and *in silico* predictions for human-relevant risk assessment

For the *in vitro* assessment of TH system disruptors, additional focus will be on the liver. This is an important area to consider because a large proportion of chemical compounds may cause TH system disruption through effects in the liver (extrathyroidal mode of action), including per- and polyfluoroalkyl substances (PFAS), flame retardants, pesticides and food additives. With attention on the developing liver, a human-relevant stem cell-based *in vitro* model will be used to assess TH-mediated liver toxicity relevant for the early phases of development. Multipotent human skin-derived precursor cells (hSKPs) will be differentiated towards “hepatic progenitor cells” (hSKP-HPC), mimicking human liver embryogenesis (De Kock et al., 2009; Snykers et al., 2009). With this assay we aim to develop a method to characterize effects of chemicals on the developing liver, TH-signaling and its downstream effects (e.g., lipid metabolism), but also how chemicals may change the TH system in the liver (including deiodination and sulfation, nuclear receptor activation, TH distribution and transmembrane transport) and their downstream consequences for the organism. To achieve this, we will for instance use transcriptomics approaches to enable comparisons with effects observed in *in vivo* toxicity studies to scrutinize potential similarities and differences between rats and humans. Finally, through synergistic activities across PARC projects, physiological-based kinetics (PBK) modelling will further aid in the quantitative understanding of causal relationships leading to adverse effect outcomes.

In addition to the *in vitro* assays, we will develop new quantitative structure–activity relationship (QSAR) models for *in silico* prediction of chemical substances’ potential TH disrupting properties. They will be developed to expand on the existing publicly available battery of endpoints relevant for TH system disruption (see e.g., www.qsar.food.dtu.dk). QSAR models that are already finalized or under development by the PARC partner include TPO, NIS, TRs, deiodinases (DIOs), pregnane X receptor (PXR), constitutive androstane receptor (CAR), and aryl hydrocarbon receptor (AhR). This project will contribute with models for TTR binding (expansion/remodeling with other technologies of previously published model (Zhang et al., 2015)) and, to our knowledge, the first models for monocarboxylate transporter 8 (MCT8) and iodothyrosine dehalogenase (DEHAL1) activity, and possibly more. Generation of additional experimental data to challenge and expand the predictive capacity of certain QSAR models is also planned as collaborative activities across the PARC partnership.

To address some of the complexities of the TH system, we will explore the use of a 3D cell culture to model the complex process of TH synthesis and develop assays to test for potential chemical interference with TH transport across biological barriers. We will explore a thyroid follicle model (human or animal) for its inherent characteristics, ability for TH synthesis and its predictive capacity for TH system disruption by comparison with results from various NAMs, e.g., covering NIS and TPO activities. We will develop *in vitro* bioassays to study the inhibition of TH transport across physiological barriers with a focus on the blood-cerebrospinal fluid-barrier. This is crucial since TH action depends on TH being actively transported into both cells, the fetus and the brain. To this end, an induced pluripotent stem cell (iPSC)-based choroid plexus organoid model capable of excreting a cerebrospinal (CSF)-like fluid (Pellegrini et al., 2020) will be developed and used to study TH transport across the blood-cerebrospinal fluid-barrier and to identify new TH transmembrane transporters. Then, relevant transporters will be selected to expand the battery of stable TH transmembrane transporter-overexpressing cell lines, where MCT8 currently exist and organic-anion-transporting polypeptides 1C1 (OATP1C1) and organic anion transporter 4 (OAT4) are under development. These cell lines can be used to screen compounds for their capacity to inhibit TH transmembrane transporters that not only play a role in TH transport across the blood-cerebrospinal fluid-barrier, but also across the placenta and the blood-brain-barrier.

### 3.3 Non-mammalian assays for human health-relevant assessment of chemicals

To further facilitate the implementation of more NAMs for human-relevant chemical risk assessment, we will assess the benefit of including non-mammalian test species such as fish embryos and invertebrates in IATAs tailored for human and ecological health parameters. Fish embryos are not protected under the current EU legislation for the use of laboratory animals until the free-feeding stage, which corresponds to 5 days post fertilization (dpf) for zebrafish kept at 28°C (European Commission, 2020). This represents an opportunity for reducing animal testing. The TH system is highly conserved across vertebrate taxa and using data from, for instance, fish to inform on potential risks to human health would greatly facilitate the transition away from high reliance on mammalian animal toxicity testing. Development of the TH system in zebrafish covers the earliest phases of embryonic development that rely on maternally transferred THs, but also the development of the entire HPT axis, which parallels human development. During the first 5 dpf the thyroid and HPT-axis develops into a fully functional organ and, importantly, is susceptible to TPO inhibition (Opitz et al., 2011; Stinckens et al., 2016; Persani and Marelli, 2017; Vergauwen et al., 2018; Walter et al., 2019). Furthermore, elements of a vertebrate-like TH signaling pathway (that is; TRs, retinoid X receptor (RXR) and enzymes involved in vertebrate TH synthesis) have been identified in some invertebrate phyla (Taylor and Heyland, 2017) including mollusks, and both TRs and TH (of endogenous and/or exogenous origin) appear to regulate aspects of larval metamorphosis in some mollusk species (Morthorst et al., 2023). Although the exact mechanisms are largely unknown, disruption of TH signaling and metamorphosis in invertebrates may inform on potential effects in vertebrates.

We will establish new assays in zebrafish to determine the function of iodide and TH transporters. We will apply genomic and proteomic approaches to zebrafish toxicity studies to decipher the modes of action and characterize the relationships between the different MIEs and their key downstream events. These molecular analyses will be correlated to behavioral assays using zebrafish larvae to assess how potential TH system disruption affects brain development and alters behavior, for instance tail coiling to assess early motor innervation (Saint-Amant and Drapeau, 1998; de Oliveira et al., 2021), locomotor response to changes in light condition to assess the integration of the central nervous system with the peripheral nervous system and sensory organs (Berg et al., 2018), anxiety-like behaviors to assess serotonergic, dopaminergic, and adrenergic system function (Markin et al., 2021), and a habituation assay in response to auditory and light stimuli to assess non-associative learning (Roberts et al., 2013). This integration will link MIEs and KEs with AOs at the level of an intact organism. Finally, functional and proteomics data from zebrafish will be compared to data from human-based *in vitro* assays to validate cross-species extrapolation capacity.

To further investigate the potential of using data from e.g., fish to inform on potential risks to human health, zebrafish embryo assay data will be used in cross-species extrapolation case studies. We will collect TH system disruption data from different vertebrate taxa (i.e., fish, amphibians and mammals), both from the literature as well as data becoming available in PARC (see work described above), over the next years. This work will use the AOP framework, and specifically the TH system disruption cross-species AOP network and the associated evaluation of the taxonomic domain of applicability, to anchor observations across species to common toxicological pathways. This will aid the development of strategies for cross-species extrapolation, and sharing knowledge between human and environmental health evaluation.

## 4 Concluding remarks

As we move towards greater reliance on NAMs for chemical risk assessment, we need to close existing knowledge gaps to ensure adequate test and non-test evaluation of TH system disrupting properties. By focusing on *in vivo* rodent toxicogenomics, advanced stem cell-based *in vitro* bioassays, transporter and zebrafish embryo investigations, concomitant with cross-species and *in silico* studies, this project, under the PARC framework, will close important gaps in knowledge and available tests necessary to facilitate further 3R approaches for a testing framework capable of adequately identifying TH system disruptors. In other words, this project aims to improve human health risk assessment of TH system disrupting chemicals to better safeguard human health and the environment.
